# A novel cuproptosis-related subtypes and gene signature associates with immunophenotype and predicts prognosis accurately in neuroblastoma

**DOI:** 10.3389/fimmu.2022.999849

**Published:** 2022-09-23

**Authors:** Xiao-Mao Tian, Bin Xiang, Yi-Hang Yu, Qi Li, Zhao-Xia Zhang, Chenghao Zhanghuang, Li-Ming Jin, Jin-Kui Wang, Tao Mi, Mei-Lin Chen, Feng Liu, Guang-Hui Wei

**Affiliations:** ^1^Department of Urology, Children’s Hospital of Chongqing Medical University, Chongqing, China; ^2^Ministry of Education Key Laboratory of Child Development and Disorders, Chongqing Key Laboratory of Pediatrics, National Clinical Research Center for Child Health and Disorders, China International Science and Technology Cooperation Base of Child Development and Critical Disorders, Children’s Hospital of Chongqing Medical University, Chongqing, China; ^3^Chongqing Key Laboratory of Children Urogenital Development and Tissue Engineering, Chongqing, China

**Keywords:** neuroblastoma, cuproptosis, tumor immune microenvironment, prognosis, immunotherapy

## Abstract

**Background:**

Neuroblastoma (NB) is the most frequent solid tumor in pediatrics, which accounts for roughly 15% of cancer-related mortality in children. NB exhibited genetic, morphologic, and clinical heterogeneity, which limited the efficacy of available therapeutic approaches. Recently, a new term ‘cuproptosis’ has been used to denote a unique biological process triggered by the action of copper. In this instance, selectively inducing copper death is likely to successfully overcome the limitations of conventional anticancer drugs. However, there is still a gap regarding the role of cuproptosis in cancer, especially in pediatric neuroblastoma.

**Methods:**

We characterized the specific expression of cuproptosis-related genes (CRGs) in NB samples based on publicly available mRNA expression profile data. Consensus clustering and Lasso-Cox regression analysis were applied for CRGs in three independent cohorts. ESTIMATE and Xcell algorithm was utilized to visualize TME score and immune cell subpopulations’ relative abundances. Tumor Immune Dysfunction and Exclusion (TIDE) score was used to predict tumor response to immune checkpoint inhibitors. To decipher the underlying mechanism, GSVA was applied to explore enriched pathways associated with cuproptosis signature and Connectivity map (CMap) analysis for drug exploration. Finally, qPCR verified the expression levels of risk-genes in NB cell lines. In addition, PDHA1 was screened and further validated by immunofluorescence in human clinical samples and loss-of-function assays.

**Results:**

We initially classified NB patients according to CRGs and identified two cuproptosis-related subtypes that were associated with prognosis and immunophenotype. After this, a cuproptosis-related prognostic model was constructed and validated by LASSO regression in three independent cohorts. This model can accurately predict prognosis, immune infiltration, and immunotherapy responses. These genes also showed differential expression in various characteristic groups of all three datasets and NB cell lines. Loss-of-function experiments indicated that PDHA1 silencing significantly suppressed the proliferation, migration, and invasion, in turn, promoted cell cycle arrest at the S phase and apoptosis of NB cells.

**Conclusions:**

Taken together, this study may shed light on new research areas for NB patients from the cuproptosis perspective.

## Introduction

Neuroblastoma (NB) is an embryonal tumor arising from the peripheral sympathetic nervous system and is the most common extracranial tumor of childhood. The age range most typically affected infants between 18- and 22- months, with most cases diagnosed before 5-years of age (1). NB originates from the precursor cells of the sympathetic nervous system, which typically present as a mass in the adrenal gland or sympathetic ganglion (2, 3). Approximately 700 children were diagnosed with NB each year in the United States, accounting for 8-10% of all childhood tumous and 15% of childhood tumor deaths (4). NB has long been recognized as one of the most mysterious cancers due to the variability of its outcome. In some cases, NB can completely degenerate or differentiate spontaneously, resulting in complete recovery without any intervention; whereas, in others, NB develop extensive metastases with very poor outcomes (5). The current standard of therapy for high-risk NB contained induction, consolidation, and maintenance (6). About half of high-risk patients failed to respond to standard treatment protocols or relapsed in the first two years after treatment, resulting in very poor outcomes, with long-term survival rates remaining less than 50% (7–10).

Recent advances in immunotherapy have contributed to a significant improvement in outcomes for various adult malignancies (11). The most sophisticated immune interventions involve immune checkpoint inhibition (ICI), antibody-mediated therapy, and adoptive T cell therapy. Increased survival of high-risk NB patients after implementation of anti-GD2 therapy demonstrates the potential of immunotherapy in pediatric oncology (12, 13). An attractive feature of the immunotherapeutic approach is its lack of long-term and accumulating toxicity, which is particularly important in pediatric organisms. However, highly heterogeneous nature of NB may require an individualized approach in which the genetic, biochemical, and phenotypic characteristics in each specific patient are evaluated individually to select the best combination therapy. Given that only a subset of patients respond to immunotherapy, identifying biomarkers that predict response to therapy is an important aspect of implementing immunotherapy in NB. These approaches will also help identify novel targets that lead to malignant transformation and progression of NB.

Very recently, Tsvetkov et al. revealed that copper toxicity involves the disruption of specific mitochondrial metabolic enzymes, thereby triggering an unusual cell death mechanism, termed ‘cuproptosis’ (14). Researchers described cuproptosis as a non-apoptotic cell death pathway. It relies on the accumulation of copper in cells, unlike all existing known ones. This work shows that copper toxicity is strongly associated with the mitochondrial activity. Significantly, to identify unique metabolic pathways for copper-mediated cytotoxicity, a genome-wide CRISPR-Cas9 loss-of-function screen was used followed by individual knockout experiments to further identify key genes that contribute to cuproptosis. This groundbreaking research opens up a refreshing pathway to cell death. Interestingly, several studies have shown that copper dysregulation in SH-SY5Y human neuroblastoma cells induces apoptosis through various pathways such as mitochondrial damage and oxidative stress (15–18). In addition, a previous pivotal study revealed that copper chelating drugs mediate PD-L1-driven cancer immune evasion (19). As a paradigm for big data research in pediatric oncology, several risk models have been identified in NB that can accurately predict prognosis (20–22). Yet, the role of cuproptosis in the immune landscape and prognosis of NB remains to be elucidated.

**Figure 1:**
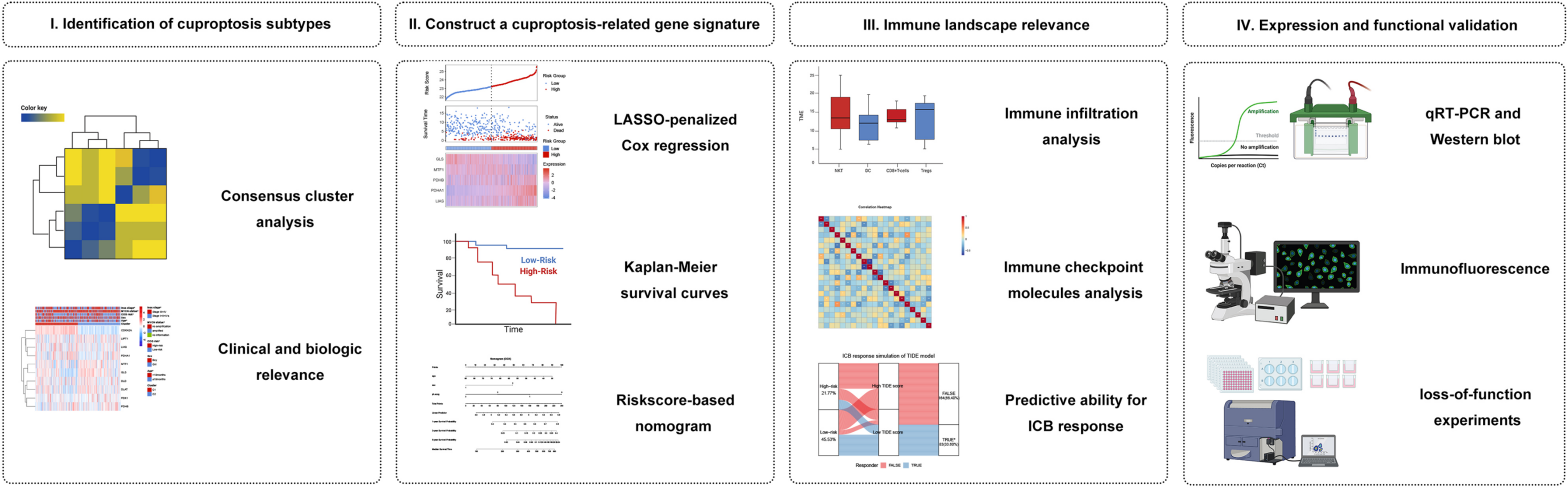
Graphical abstract. **(I)** Identification and comprehensive analysis of cuproptosis subtypes in NB. **(II)** Construction and validation of the cuproptosis signature in NB. **(III)** Association with immune infiltration, immune checkpoint molecules, and its predictive ability for the response to immunotherapy. **(IV)** Expression Validation and Functional Validation.

We hypothesized that cuproptosis-related gene signature allows for a valuable prognosis biomarker and allows precise TIM (Tumor immune microenvironment) characterization in NB patients. Here, we initially characterized the specific expression of cuproptosis-related genes (CRGs) in NB samples based on publicly available mRNA expression profile data. We classified NB patients in the GEO cohort according to CRGs and identified two cuproptosis-related subtypes that were associated with prognosis and immunophenotype. Subsequently, a cuproptosis-related prognostic signature was constructed and validated by LASSO regression. This model can accurately predict prognosis, immune infiltration, and immunotherapy response. This study may shed light on new research areas for NB patients from the cuproptosis perspective. **Figure 1** shows a schematic representation of the major steps to portray our study clearer.

## Materials and methods

### NB dataset source, preparation, and processing

Gene expression data with relevant complete clinicopathologic variables were collected from the publicly available ArrayExpress database, GEO database, and TARGET database. In total, we obtained 3 independent datasets including GSE49711 cohort from GEO database, TARGET-NB cohort from TARGET database, and E-MTAB-8248 cohort from ArrayExpress database. Patients with unavailable follow-up information were excluded, and 968 patients were enrolled for subsequent data analyses ultimately. Relevant clinical variables included age, sex, race, ploidy, COG risk, histology, MKI, 1p del/im, alt status, MYCN status, Inss stage, and survival information. Given the maximum effective sample size, we selected the GSE49711 cohort to serve as the ‘training cohort’ in which a prognosis gene signature was developed. Subsequently, the remaining two datasets, TARGET-NB (phs000467) and E-MTAB-8248 cohort (23) were used as the ‘verification cohort’ to test the reliability and immune correlation of the signature. More details of these patients are provided in **Supplementary Table 1**. All of analyses data are publicly available and extracted from open data platforms, no ethics approval or patient consent was required. The study was conducted in full compliance with the publication requirements from TARGET, GEO, and ArrayExpress.

### Consensus clustering analysis for cuproptosis-related genes (CRGs)

10 CRGs were retrieved from the recent publication (14), as previously described. Based on the expression profile of the 10 CRGs, 498 NB patients from GSE49711 were classified using the unsupervised clustering analysis. ConsensusClusterPlus (24) was used to perform cluster analysis, which included agglomerative pam clustering with 1-pearson correlation distances and resampling 80 percent of the samples for 10 times. The empirical cumulative distribution function graph was used to identify the optimized number of clusters. Then, the principal component analysis (PCA) for the two subtypes was constructed using the ‘scatterplot3d’ R packages in terms of gene expressions of 10 CRGs. We next evaluated the correlations between molecular subtypes, clinicopathological features, and prognosis to determine the clinical utility of the two subtypes established by consensus clustering. Gene set variation analysis (GSVA) (25) was used with the hallmark gene set (c2.cp.kegg.v7.4) obtained from the MSigDB database (26) to explore the diversity of CRGs in biological pathways. The R package ‘limma’ (27) was used to analyze differences for functional annotation. Significant variations in KEGG pathways were defined as P values less than 0.05.

### Establishment of a cuproptosis-related gene signature

We used data from GSE49711 cohort as our training cohort. Initial screening for prognosis-related CRGs was performed by using univariate Cox regression analysis. We focused on prognosis-related CRGs to construct a prognostic cuproptosis gene signature. The Cox proportional hazards model with a Lasso penalty (iteration = 10) was used to discover the optimal gene model for the CRGs with prognostic ability, using the R package ‘glmnet’ (28). The cuproptosis signature was constructed by combining the selected gene expression levels in a linear fashion and weighting them according to their Lasso-Cox regression coefficients. Riskscore = ∑in(Coefi * Xi) depicted the developed prognostic model succinctly, where X represented the expression level of each IRG and Coef represented the coefficient of relative prognostic IRGs in the model. This formula was applied to calculate each patient’s riskscore, and the median score was defined as a cut-off value between high-risk and low-risk subgroups. Also, PCA for the two subgroups was constructed using the ‘scatterplot3d’ R packages in terms of the risk genes expressions. In addition, Kaplan-Meier survival curves and time-dependent receiver operating characteristic (ROC) curves were performed to assess the predictive power of this signature.

### Validation of the cuproptosis signature

In the validation phase, two independent datasets were obtained to validate the cuproptosis-associated riskscore model through the TARGET database (TARGET-NB cohort) and ArrayExpress database (E-MTAB-8248 cohort). To verify the clinical value of the cuproptosis signature, the distribution of clinicopathological features was also assessed in the two risk subgroups analyzed by chi-square test and visualized with heatmaps. Furthermore, to verify whether the predictive power of cuproptosis signature was independent of conventional clinical characteristics, univariate and multivariate Cox regression and stratified analysis was conducted. Subsequently, a nomogram was built using the abovementioned variables *via* the Cox proportional hazards model. Finally, ROC curves, calibration curves, and the decision curve analysis (DCA) performed by the R package ‘rmda’ were used to measure the accuracy of the nomogram.

### Relationship between cuproptosis signature and TIM

The StromalScore, ImmuneScore, and ESTIMATEScore, that reflect the TME-related cell infiltrating degree in tumor tissues of NB were estimated by R package ‘IOBR’ using the ESTIMATE algorithm (29, 30), which was based on the single sample gene set enrichment analysis (ssGSEA). Tumors are highly heterogeneous tissues in which the tumor microenvironment (TME), which contains a variety of immune cell types, surrounds and interacts with malignant cells. To assess the heterogeneous cellular landscape of TME, the enrichment fraction of cell types was evaluated. We used the Xcell algorithms (31), an R package for cell type enrichment analysis of 64 cells in TME based on gene expression profile, to quantify the correlated abundance of immune-cell infiltrations in tumor samples through ssGSEA. Then, we compared the difference in infiltrating immune cells in the high- and low-risk subgroups by using the two-sample Wilcoxon test. To explain the differences in survival of NB patients from an immune perspective, we applied survival analysis to compare the differences based on the Stromal, Immune, ESTIMATE-scores and the fraction of immune cell infiltration in the TME of NB patients.

### Relationship between the cuproptosis signature and ICB immunotherapy

We further analyzed the association with immune checkpoint molecules of the gene signature and its effect on the potential response to immune checkpoint blockade (ICB) therapy in the TARGET-NB cohort. 57 immune checkpoint molecules, including 22 inhibitory- and 35 stimulatory-immune checkpoint genes, were identified as immune checkpoint-relevant transcriptions. The correlation between riskscore and immune checkpoint genes’ expression was calculated by Pearson correlation. Additionally, the Tumor Immune Dysfunction and Exclusion (TIDE) score, generated using a computational algorithm on the basis of the corresponding gene expression profile, was used to predict tumor response to immune checkpoint inhibitors (32). In brief, the higher the TIDE score, the worse the treatment response and outcomes.

### Mechanism exploration and candidate small molecule drugs

To assess the potential difference in biological pathways between the high- and low-risk subgroups, we used GSVA analysis to explore significantly enriched signaling pathways. The connectivity map (cMap) database (https://clue.io/) is unravel biology with the world’s largest perturbation-driven gene expression dataset. We discovered predicted drugs that may aggravate or avoid the biological processes of tumors according to the up-regulated and down-regulated genes when comparing the high-risk and low-risk subgroups. With an FDR value of less than 0.05 and an enrichment score ranging between -1 and 0, the prospective drugs could be served as a novel target candidate for NB patients. These putative drugs’ 3D structural images were acquired from the PubChem database (https://pubchem.ncbi.nlm.nih.gov/).

### qRT-PCR assay

The expression of Riskgenes in the NB cell lines (SH-SY5Y and BE(2)-C) was verified *via* qRT-PCR assay. Detailed experimental protocol refers to previously published literature (33). The primers used are listed in **Supplementary Table 5A**.

### Immunofluorescence

Of these Riskgenes, PDHA1 plays a crucial role in multiple malignancies as a tumor suppressor or oncogene. We detected the expression pattern of PDHA1 (1:500, Abcam) in human clinical samples using immunofluorescence, which was carried out as we described previously (34).

### Loss of function experiments

BE(2)-C cells were transfected with PDHA1 siRNAs (Tsingke, China) for loss-of-function experiments (The sequences used for PDHA1 silencing are shown in **Supplementary Table 5B**). PCR and western blotting were performed to determine the silencing efficiency of PDHA1. CCK8 assay (MCE, HY-K0301), scratch wound healing assay, and Transwell assay (Falcon, 353097, USA and Biozellen, B-P-00002-4, China) were investigated to detect cell viability, cell migration, and cell invasion according to the manufacturer’s instructions. Cell cycle and apoptosis assay were carried out by flow cytometry using the BD detection kit.

### Statistical analysis

GraphPad Prism version 8 software (GraphPad Prism Software Inc., La Jolla, CA) was used to analyze the experimental results. R software was used for all bioinformatics analyses and R packages. The significance level is indicated by single, double, and triple asterisks, as well as ns (*, **, ***, and **** indicated a significance level of 0.05, 0.01, 0.001, and 0.0001 respectively; and ns indicates no significant level).

## Results

### Identification and comprehensive analysis of cuproptosis subtypes in NB

**Figure 2:**
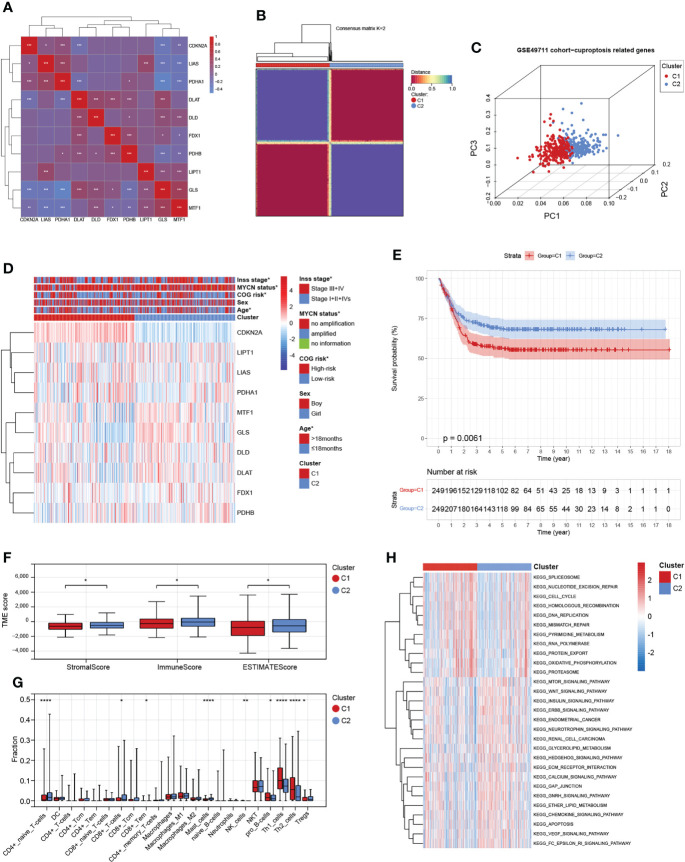
Identification and comprehensive analysis of cuproptosis subtypes in NB. **(A)** Expression-related clustering heat map of 10 cuproptosis-related genes. **(B)** Consistency of clustering results in heatmap (k = 2). Rows and columns represent samples, the different colors represent different types. **(C)** PCA analysis shows a remarkable difference in transcriptomes between the two subtypes. **(D)** Heatmap of the clinical correlation between the two subtypes in NB. **(E)** Patients in cluster C1 exhibited worse progression-free survival compared to those in cluster C2. **(F)** Estimatescore, Immunescore, and Stromalscore in the two subtypes. **(G)** Distribution of 22 types of immune-infiltrating cells in the two subtypes. **(H)** GSVA of biological pathways between the two distinct subtypes. *, **, ***, and **** indicate a significance level of 0.05, 0.01, 0.001, and 0.0001, respectively.

We obtained 10 key cuproptosis-related genes (CRGs) from recently published significant findings. A Spearman correlation analysis confirmed a varying degree of association between these gene expressions in NB (**Figure 2A**). To interrogate the expression importance of CRGs in NB, we focused on the expression profile of the 10 CRGs and clustered NB patients using the consensus Clustering algorithm. The results indicated for K = 2 was the optimum K value, suggesting that we could divide the patients into two groups (**Supplementary Figures 1A-D**). Thus, two genetically distinct subtypes of NB have been defined, subtype C1 and subtype C2, respectively (**Figure 2B**). The clear difference in CRGs was further demonstrated by principal components analysis (PCA, **Figure 2C**). We next compared the clinicopathological characteristics between the two molecular subtypes. Multiple clinicopathological features, including age, COG risk, MYCN status, and Inss stage were significantly different between the two subtypes (**Figure 2D**). Furthermore, Kaplan-Meier curves indicate that individuals with subtype C1 had significantly worse RFS (**Figure 2E**). To investigate the role of CRGs in the immune microenvironment in NB, we evaluated the TME score and immune cell infiltration using a computational algorithm, ESTIMATE, and Xcell, on transcriptional profiles of the GSE49711 cohort (**Supplementary Tables 2** and **3**). Higher stromal or immune scores represent higher relative levels of stromal or immune cells in the TME. We observed a relatively higher StromalScore, ImmuneScore, and ESTIMATEScore in subtype C2 (**Figure 2F**). For immune cell infiltration, the infiltration fraction of CD4^+^ naive T cells, CD8^+^ Tcm, CD8^+^ Tem, Mast cells, and Tregs in subtype C2 was significantly higher than those in subtype C1, while NK cells, pro B cells, Th1 cells, and Th2 cells in subtype C2 had lower infiltration compared to subtype C1 (**Figure 2G**). This may in part explain the observation that subtype C2 confers a survival advantage compared to subtype C1. These results also demonstrate an essential role of CRGs in TME of NB. GSVA analysis indicated that subtype C1 was significantly enriched in cell cycle-related processes, including spliceosome, nucleotide excision repair, cell cycle, homologous recombination, DNA replication, and mismatch repair, and subtype C2 were mainly enriched in carcinogenic pathways, such as the MTOR, WNT, INSULIN, CHEMOKINE, and VEGF signaling pathways (**Figure 2H**).

### Construction and validation of the cuproptosis signature

**Figure 3:**
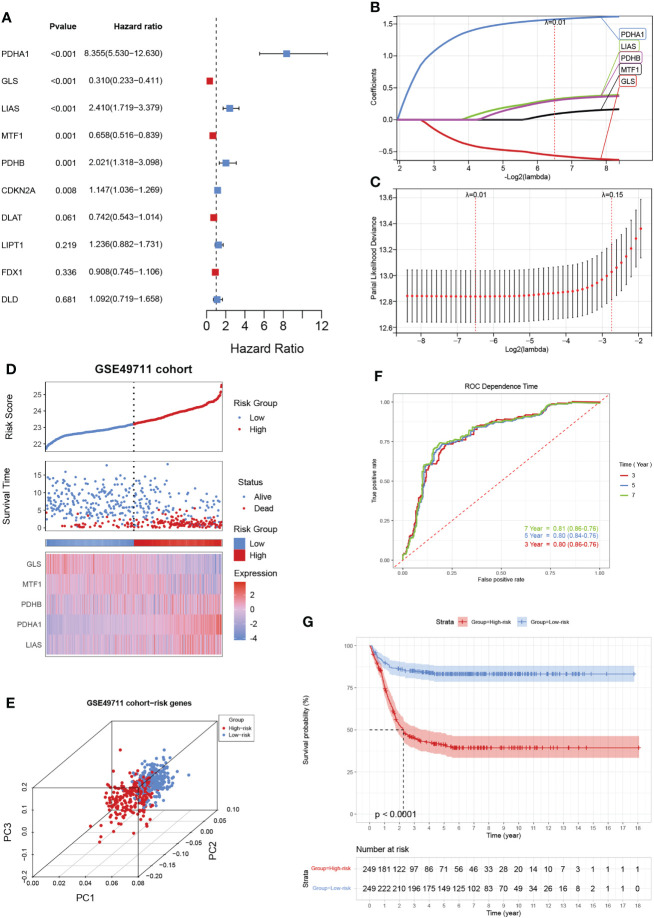
Construction of a cuproptosis-related gene signature in the training cohort. **(A)** The forest map shows six genes significantly correlated with progression-free survival in the univariable Cox regression analysis. **(B)** The trajectory of each independent variable. The horizontal axis represents the log value of the independent lambda, and the vertical axis represents the coefficient of the independent variable. **(C)** Partial likelihood deviance of variables revealed by the Lasso regression model. The red dots represented the partial likelihood of deviance values, the gray lines represented the standard error (SE), and the two vertical dotted lines on the left and right represented optimal values by minimum criteria and 1-SE criteria, respectively. **(D)** Distribution of the riskscore, the associated survival data, and the mRNA expression heatmap of the gene signature in the GSE49711 cohort. Patients were divided into high-risk (red) and low-risk (blue) groups and the median risk score was utilized as the cutoff value. **(E)** PCA revealed a clear separation between the high- and low-risk subgroups, based on the expression of these 5 risk genes. **(F)** The time-dependent ROC curves were applied to assess the predictive performance of the cuproptosis-related gene signature, in 3-, 5-, and 7-year survival. **(G)** Patients in the high-risk subgroup exhibited worse survival compared to those in the low-risk subgroup in the GSE49711 cohort.

Given the largest effective sample size in the GSE49711 cohort, we chose this dataset as a discovery cohort to identify a cuproptosis signature. Univariate analysis using the Log-rank test showed six CRGs with a prognostic ability (**Figure 3A**). We next used LASSO Cox regression analysis to identify the optimal values of the penalty parameter and establish the most optimal prognostic signature. A coefficient profile plot was generated against the log λ sequence, for which the optimal λ led to five nonzero coefficients (**Figures 3B, C**). A cuproptosis-related five-gene model that reached an optimal prediction efficiency was ultimately obtained. Then, five-gene cuproptosis signature was constructed using the independent regression coefficients of each gene, and the riskscore was calculated as (1.573)*PDHA1 + (-0.561)*GLS + (0.320)*LIAS + (0.088)*MTF1 + (0.301)*PDHB. Subsequently, based on the above riskscore formula, the riskscore of each patient was measured, and the patients were separated into a high- or low-risk subgroup based on their riskscore. Gene expressions in the signature lists of each patient were visualized using a heatmap (**Figure 3D**). PCA revealed a clear separation between the two risk subgroups, based on the expression of these 5 risk genes (**Figure 3E**). The time-dependent ROC curves were applied to assess the predictive accuracy of the cuproptosis-related gene signature, and the area under the curve (AUC) values predicting 3-, 5-, and 7-year survivals were 0.80, 0.80, and 0.81, respectively (**Figure 3F**). Finally, as expected, NB patients in the high-risk subgroup had dramatically worse survival than those in the low-risk subgroup in the GSE49711 cohort (**Figure 3G**).

**Figure 4:**
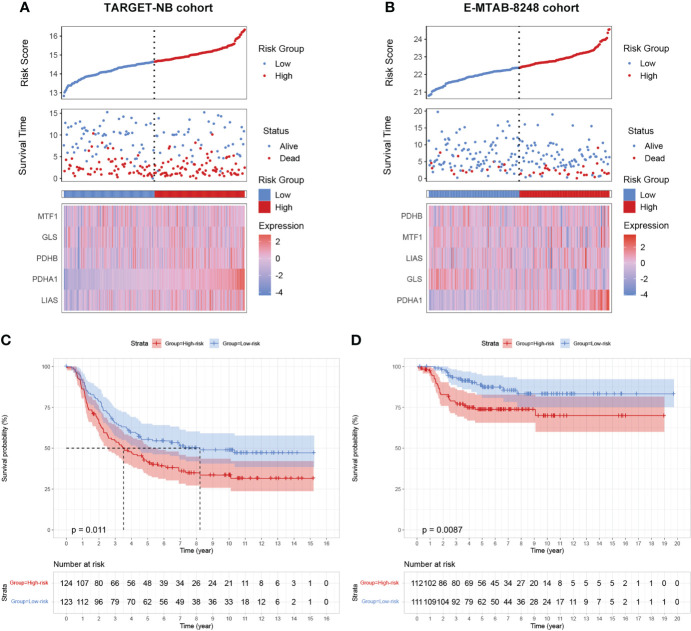
validation of the cuproptosis signature in two independent NB cohorts. **(A, B)** Distribution of the riskscore, the associated survival data, and the mRNA expression heatmap of the gene signature in the TARGET-NB and E-MTAB-8248 cohort. **(C, D)** Patients in the high-risk subgroup exhibited worse survival compared to those in the low-risk subgroup both in the TARGET-NB and E-MTAB-8248 cohort.

To further validate our prognostic model’s applicability in NB patients, we applied the prognostic model established in the GSE49711 cohort to the two other independent NB cohorts. Subsequently, we calculated the riskscore of each patient in the TARGET-NB and E-MTAB-8248 cohort based on the developed risk model and then plotted the riskscore distribution (**Figures 4A, B**). Depending on the corresponding median score, the patients were stratified into a high- and low-risk subgroup. As expected, survival analysis demonstrated that patients in the high-risk subgroup have a worse prognosis than those in the low-risk subgroup, both in the TARGET-NB and E-MTAB-8248 cohort (**Figures 4C, D**).

### Association with clinicopathologic factors and construction of the nomogram and its accuracy verification

**Figure 5:**
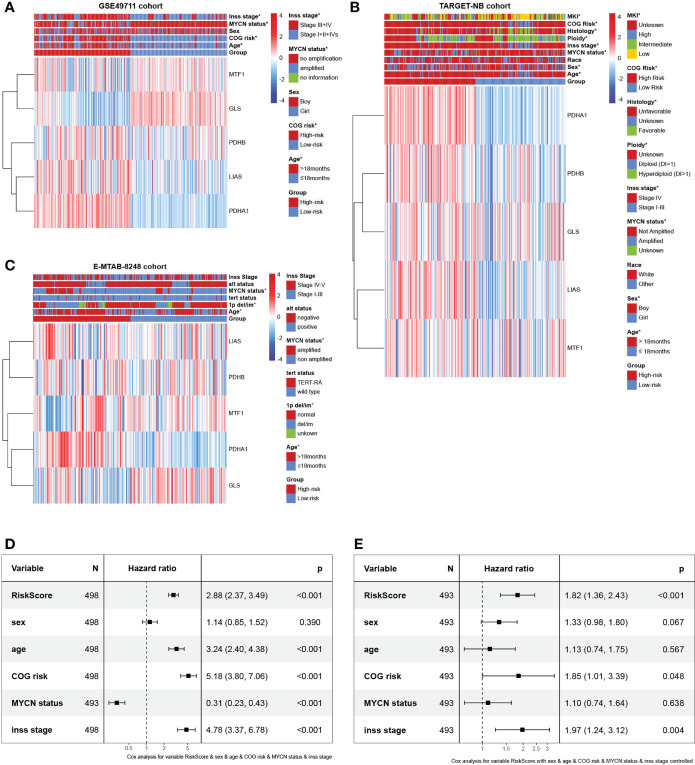
Prognostic value and clinical relevance of the cuproptosis-related gene signature. **(A–C)** Relationships between clinicopathologic features and the two risk subgroups in the GSE49711, TARGET-NB, and E-MTAB-8248 cohort. **(D, E)** The univariate and multivariate Cox regression analysis of risk factors in NB in the GSE49711 cohort.

To investigate the clinical value of the signature in NB patients, we assessed the relationship between the cuproptosis signature and clinicopathologic features. Here, we plotted a composite heat map to display the correlations of risk groups and clinicopathologic factors (**Figures 5A–C**). Between the high- and low-risk subgroups, the difference in age, COG risk, MYCN status, and Inss stage in the GSE49711 cohort, difference in age, sex, MYCN status, Inss stage, ploidy, histology, COG risk, and MKI in the TARGET-NB cohort, and difference in age, 1p del/im, and MYCN status in the E-MTAB-8248 cohort were statistically significant (P < 0.05).

Next, we scrutinized its value for predicting clinical outcomes in NB patients. In univariate Cox proportional hazards analysis in the GSE49711 cohort, the riskscore of this signature was significantly associated with patients’ RFS (**Figure 5D**). In multivariate Cox regression analysis, after adjusting for the traditional clinical prognostic variables (age, sex, COG risk, MYCN status, and Inss stage), the cuproptosis signature remained independently significant as a predictor of patients’ outcomes, indicating that our model was not affected by clinical features and had stability (**Figure 5E**). To explore the effect of the cuproptosis signature on prognosis in different subgroups with NB, we performed a subgroup survival analysis. This signature’s resolving capacity for prognosis remained consistently stable in different subgroups classified by age, sex, and Inss stage (**Supplementary Figures 2A-J**).

**Figure 6:**
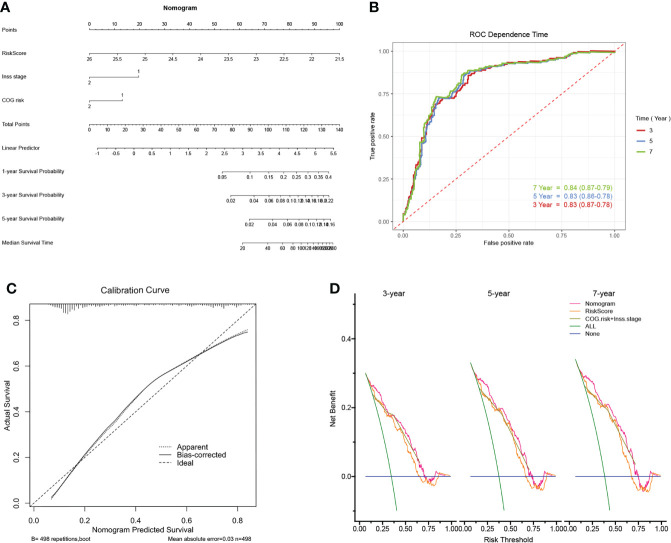
Construction of the nomogram and its accuracy verification. **(A)** The riskscore assessment nomogram to evaluate prognosis in NB (1-, 3-, and 5-year survival rates). **(B)** The time-dependent ROC curves were applied to assess the predictive performance of the nomogram, in 3-, 5-, and 7-year survival. **(C)** Calibration curves of the nomogram. **(D)** Net benefit (y-axis) as calculated are plotted against the threshold probabilities of patients having 3-, 5- and 7-year survival on the x-axis. The green line represents the assumption that all patients have indicated survival time.

For the prognostic capability of clinical indicators, the prognosis analysis from GSE49711 cohort indicated that only COG risk and Inss stage served as independent prognostic indicators for NB patients (**Figure 5E**). Together with the risk model and clinical features above, we constructed a nomogram to expand availability for clinical applications (**Figure 6A**). We assigned a riskscore to each patient by adding the points for each risk factor present, and a higher total score corresponds to a poor survival outcome. Notably, the prediction accuracy could be further improved using the full model that included both the signature riskscore and clinical prognostic factors (**Supplementary Figure 2K**). The AUC values predicting 3-, 5-, and 7-year survivals were increased to 0.83, 0.83, and 0.84, respectively (**Figure 6B**). The calibration curve (**Figure 6C**) showed well performance of the predictive model. Moreover, the DCA showed that the nomogram has favorable clinical utilization (the C-index of the nomogram for RFS was 0.736), and a more net benefit was gained from the combined nomogram model compared with the signature alone or clinical model alone (**Figure 6D**).

### Associations between the cuproptosis signature and immune infiltration in TME

**Figure 7:**
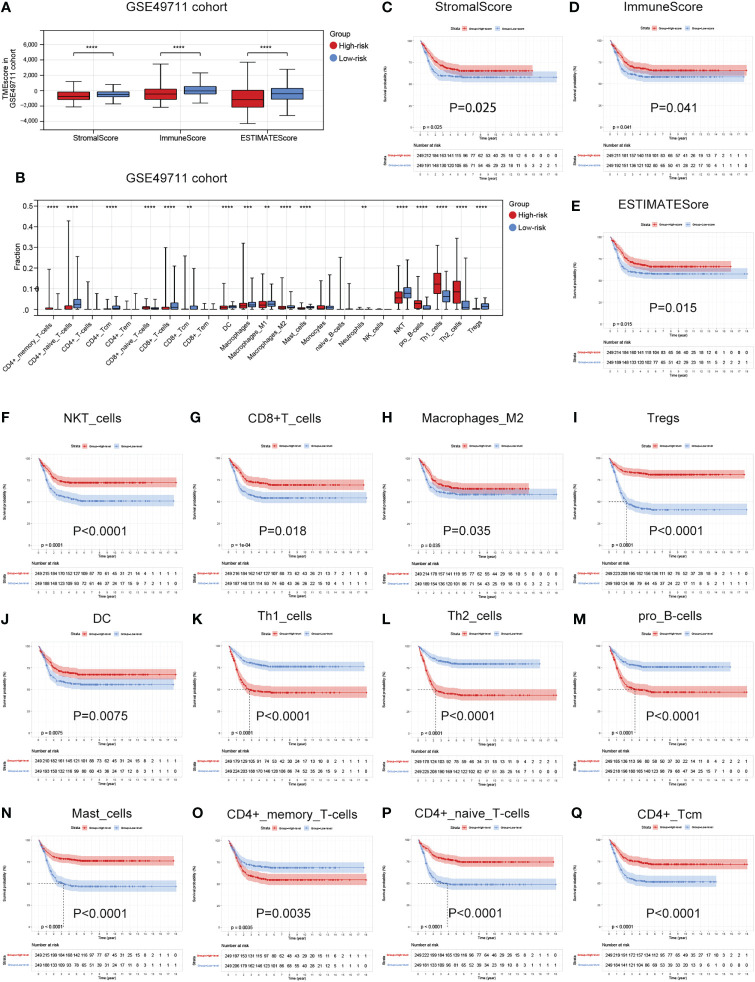
Associations between the cuproptosis signature and immune infiltration in TME. **(A)** Estimatescore, Immunescore, and Stromalscore in the two risk subgroups in the GSE49711 cohort. **(B)** Distribution of 23 types of immune-infiltrating cells in the two risk subgroups in the GSE49711 cohort. **(C–E)** Patients with a lower Estimatescore, Immunescore, or Stromalscore had a worse prognosis in the GSE49711 cohort. **(F–Q)** Patients with a lower tumor immune cell infiltration, that were differentially presented in the high- and low-risk subgroups, had a worse prognosis in the GSE49711 cohort.

As the difference in cuproptosis subtypes is closely related to the immune characteristics in TME in NB, we hypothesized that the cuproptosis signature could reflect the landscape of immune infiltration. Thus, ESTIMATE and Xcell algorithm were utilized to visualize TME score and immune cell subpopulations’ relative abundances in the GSE49711 cohort by ssGSEA (**Figure 5A**). Of greatest concern, compared with the low-risk subgroup, the high-risk subgroup had a lower StromalScore, ImmuneScore, and ESTIMATEScore (**Figure 7A**). In addition, main lymphocyte subsets involved in anti-tumor immunity, including CD4^+^ memory T cell, CD4^+^ Tcm, CD8^+^ T cell, DC, Macrophages, Mast cell, NKT cell, and Tregs were significantly increased in the low-risk subgroup (**Figure 7B**).

To explain the survival differences found in NB patients from a perspective of tumor immune, we next further assessed the association of differentially presented TME scores or immune cell infiltration abundances with survival in NB in the GSE49711 cohort. As might be expected, patients with a lower TME score and tumor immune cell infiltration, that were differentially presented in the high- and low-risk subgroups, had a worse prognosis as presented in **Figures 7C–Q**. Additionally, we performed the same analysis in the remaining two independent cohorts, and the finding was independently confirmed (Details in **Supplementary Figure 3** and **Supplementary Figure 4**). These results suggest that a favorable prognosis may in part be attributed to the activity of anti-tumor immunity.

### Associations between the cuproptosis signature and ICB immunotherapy

**Figure 8:**
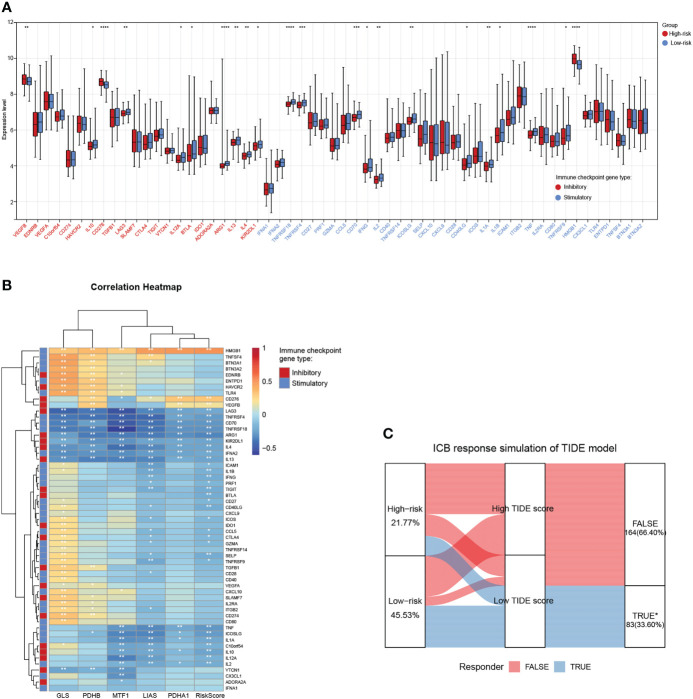
Associations between the cuproptosis signature and ICB immunotherapy. **(A)** Distribution of 57 immune checkpoint molecules, including 22 inhibitory- and 35 stimulatory-immune checkpoint genes in the two risk subgroups in the TARGET-NB cohort. **(B)** Relationships between the five risk genes, riskscore, and immune checkpoint molecules. **(C)** Immune response difference between the high- and low-risk subgroups based on TIDE scores in TARGET-NB cohort. *, **, ***, and **** indicate a significance level of 0.05, 0.01, 0.001, and 0.0001, respectively.

Tumor cells could evade immune surveillance and develop through a variety of mechanisms, including the overexpression of inhibitory immune checkpoint molecules, which inhibit anti-tumor immunological responses (35). ICB therapy has emerged as a revolutionary immune-based cancer therapy. Immune checkpoint expression might provide clues as to clinical response to immunotherapies targeting immune checkpoints. Here, the expression patterns of 57 immune checkpoint molecules, including 22 inhibitory- and 35 stimulatory-immune checkpoint genes were presented and compared in the high- and low-risk subgroups in TARGET-NB cohort. This indicated the differential expression of several inhibitory- and stimulatory-immune checkpoints such as VEGFB, IL10, CD276, LAG3, IL12A, BTLA, ARG1, IL13, IL4, KIR2DL1, TNFRSF18, TNFRSF4, CD70, IFNG, IL2, ICOSLG, CD40LG, IL1A, TNF, and HMGB1 (**Figure 8A**). Further analyses exhibited a tight correlation between the five risk genes, riskscore and immune checkpoint molecules (**Figure 8B**). Next, based on simulations of tumor immune escape mechanism, we used the TIDE algorithm to predict the response to immunotherapy in TARGET-NB cohort. Surprisingly, the results revealed that the TIDE score of patients in high-risk subgroup was higher than that in the low-risk subgroup, showing a lower response rate to ICB treatment (21.77% vs 45.53%, **Figure 8C**). These results provide further evidence that low-risk patients have better prognoses and hold a greater potential for immunotherapy applications.

### Biological pathways related to the cuproptosis signature and small molecule drugs exploration

**Figure 9:**
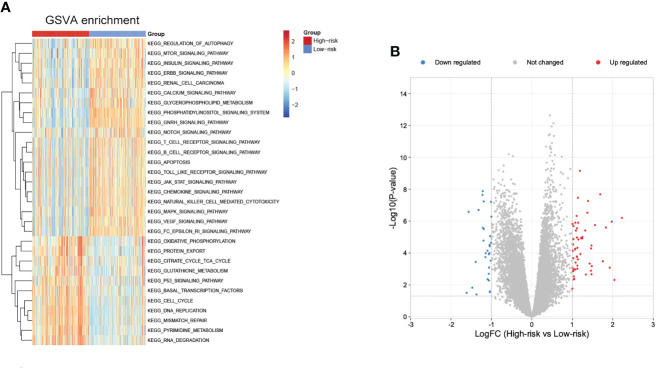
Biological pathways related to the immune signature and small molecule drugs exploration. **(A)** GSVA of biological pathways between the two risk subgroups. **(B)** Differentially expressed genes between the two risk subgroups.

To further explore potential biological pathways enriched by the cuproptosis signature, we performed GSVA analysis (**Figure 9A**). The low-risk subgroup exhibited enrichment in pathways associated with immune activation, including JAK-STAT, TOLL-like, Natural killer cell mediated cytotoxicity, chemokine, B cells and T cells receptor signaling pathways, and several carcinogenic pathways, such as mTOR, INSULIN, ERBB, NOTCH, MAPK, and VEGF signaling pathways. The high-risk subgroup was enriched in basal metabolism and ‘cell fate’-related processes, such as oxidative phosphorylation, citrate cycle TCA cycle, glutathione metabolism, and pyrimidine metabolism pathways, and protein export, DNA replication, mismatch repair, RNA degradation, and cell cycle pathways.

Moreover, small molecule drugs were explored for NB using the cMap database. 55 up-regulated and 30 down-regulated genes were identified in the comparison of low- and high-risk subgroups (**Figure 9B**). The 11 most relevant drugs were then explored as prospective candidates for NB patients based on differentially expressed genes (**Supplementary Table 4**). The 3D structures of these drugs were displayed through the PubChem database (**Supplementary Figure 5**).

### Riskgenes exhibit tissue-specific expression patterns in various groups of clinicopathological characteristics

**Figure 10:**
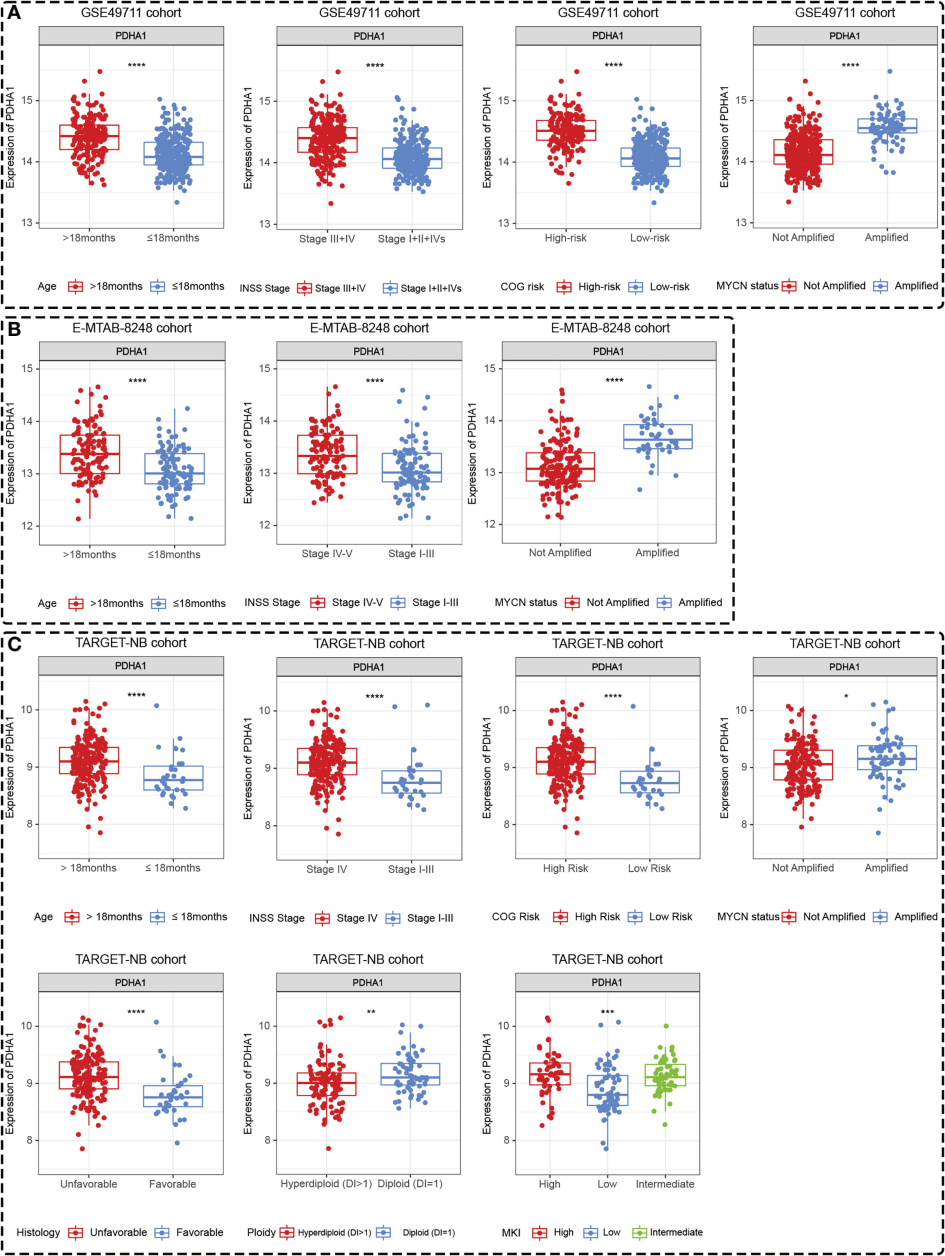
PDHA1 presented tissue-specific expression patterns in all three datasets. PDHA1 presented tissue-specific expression patterns in terms of age, Inss stage, COG risk, and MYCN status in GSE49711 dataset **(A)**, in terms of age, Inss stage, and MYCN status in E-MTAB-8248 dataset **(B)**, and in terms of age, Inss stage, COG risk, MYCN status, histology, ploidy, and MKI in TARGET-NB dataset **(C)**. *, **, ***, and **** indicate a significance level of 0.05, 0.01, 0.001, and 0.0001, respectively.

Differences in the expression levels of five Riskgenes were significant between groups in terms of clinical characteristics, including age, COG risk, Inss stage, MYCN status, histology, ploidy, and MKI (**Supplementary Figure 6** and **Supplementary Figure 7**). Moreover, PDHA1 presented significant differential expression in different clinical subgroups in three independent cohorts including GSE49711, TARGET-NB and E-MTAB-8248 cohort (**Figures 10A–C**). As presented in the discussion, this gene serves as a prognostic marker and therapeutic target for a variety of tumors. Therefore, this gene was selected for further functional analysis.

### Expression validation and functional validation

**Figure 11:**
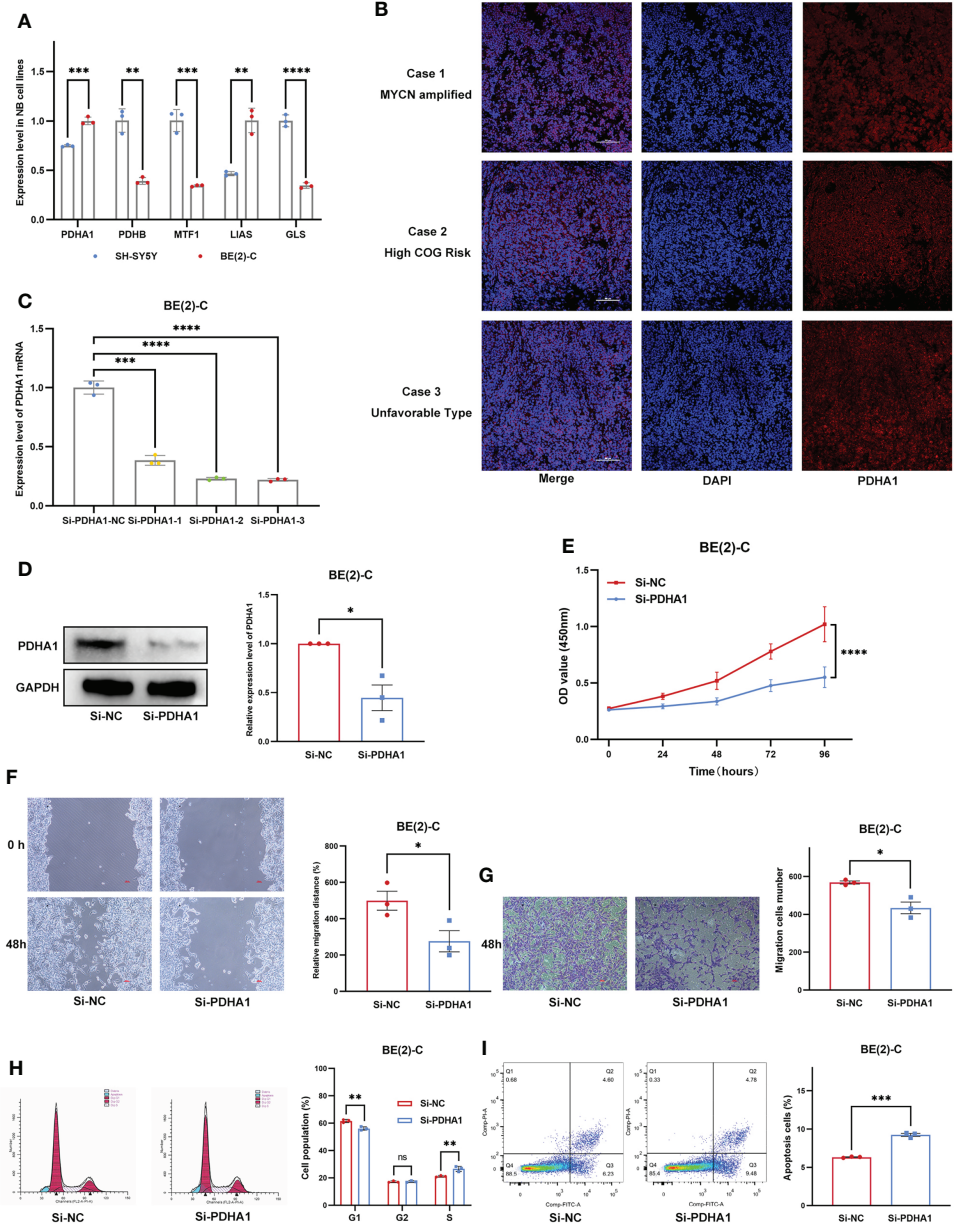
Expression Validation and Functional Validation. **(A)** Expression of five Riskgenes between SH-SY5Y and BE(2)-C cells. **(B)** Expression of PDHA1 protein in clinical samples. **(C)** qRT-PCR analysis of PDHA1 expression in NB cells after transfection with siRNAs. **(D)** Western blot analysis of PDHA1 expression in NB cells after transfection with siRNA-3. **(E–G)** Silencing of PDHA1 suppressed proliferation, invasion, and migration in NB cells. **(H,I)** Silencing of PDHA1 promoted cell cycle arrest at the S phase and apoptosis in NB cells. *, **, ***, and **** indicate a significance level of 0.05, 0.01, 0.001, and 0.0001, respectively.

We first examined expression differences of five Riskgenes between SH-SY5Y (nonamplified MYCN) and BE(2)-C (amplified MYCN) cell line (**Figure 11A**). We then examined the expression of PDHA1 protein in clinical samples, given its important role in malignancies (**Figure 11B**). As previously described, PDHA1 was highly expressed in amplified MYCN cell lines and tumor tissues. To investigate the functional role of PDHA1 in NB carcinogenesis, we designed three siRNA for silencing this gene. We chose siRNA-3 for the follow-up experiments as it is the most efficient siRNA (**Figure 11C**). Compared with the negative control, siRNA-3 greatly decreased the protein level in BE(2)-C cells (**Figure 11D**). Loss-of-function experiments showed that silencing of PDHA1 significantly suppressed the proliferation, migration and invasion ability of NB cells through CCK-8 assay, wound healing assay, and transwell assay, respectively (**Figures 11E–G**). In addition, flow analysis indicated that PDHA1 gene silencing promoted cell cycle arrest at the S phase and apoptosis of NB cells (**Figures 11H, I**).

## Discussion

For well over a century, researchers have noted that NB exhibits a diverse and dramatic clinical presentation. A subset of tumors resolved on their own, while others persistently progressed (36). The identification of patient subgroups with different biological and genetic characteristics has allowed for a refinement of risk stratification. Since the initial discovery of MYCN, many prognostic biomarkers have been proposed for NB, with the most intensive studies including histopathologic classification, tumor staging, MYCN amplification, tumor cell DNA index (ploidy), and segmental chromosomal aberrations (37–39). Evidence-based risk stratification for NB has made significant advances in the outcomes, but the prognoses of patients with high-risk NB still have a margin of significant improvement. Genetic and molecular profiling of NB using microarray, RNA-seq, or other techniques is increasingly being used to identify genetic features that predict patient prognosis. In addition, the use of molecularly targeted therapies focused on genetic abnormalities and impacted pathways provided a novel strategy for the therapy of NB patients (40). Very recently, a new term ‘cuproptosis’ has been used to denote a new form of cell death triggered by the action of copper, revealing that copper toxicity derives from the disruption of certain mitochondrial metabolic enzymes, resulting in a unique cell death mechanism (14). The mechanism could explain the pathology in relation to copper overload diseases and propose a novel way for cancer treatment (41). The utilization of metabolic features of cancer cells that can selectively induce cuproptosis is promising to overcome the limitations of conventional anti-cancer drugs. In this context, the research of cuproptosis for novel cancer therapeutic techniques is quite appealing. Significantly, the team has identified key regulatory genes that promote copper-induced cell death. As an initial aim of this study, we aimed to provide a comprehensive analysis of the role of cuproptosis-related genes in NB, which has potential clinical implications for prognostic prediction and molecular targeted drug developing.

A recent outstanding review systematically discusses the selectivity of copper ion carriers for tumor cells and the mechanisms responsible for this selectivity (42). The development of new therapeutic agents could improve selectivity and thus reduce side effects. Currently, cuproptosis has not been systematically researched in cancer. Here, we started the present study with cuproptosis mechanism, focusing on 10 key genes associated with cuproptosis in NB. In the present study, we found that NB could be divided into two independent subtypes based on the expression of 10 CRGs. The two subtypes were associated with several key clinicopathological features (age, COG risk, MYCN status, and Inss stage) and showed differences in prognosis. Despite recent advances in immunotherapy, the prognosis of NB patients continues to exhibit heterogeneity, highlighting the critical role of TME in NB tumorigenesis and progression. Therefore, we explored the immune characteristics of different subtypes. We found that TME scores and the relative abundance of several key tumor-infiltrating immune cells (CD4^+^ naive T cells, CD8^+^ Tcm, CD8^+^ Tem, Mast cells, and Tregs, NK cells, pro B cells, Th1 cells, and Th2 cells) showed significant differences between the two subtypes. These results may partially explain the observation of different prognostic characteristics of the two subtypes. It also demonstrates the important role of CRGs in TME of NB. To analyze the reasons for these differences, GSVA analysis was performed, and it was clear from the results that cluster C1 was significantly enriched for processes concerning the ‘cell cycle’, while cluster C2 was predominantly enriched for cancer-promoting signaling pathways.

Previous studies have provided clues to the potential role of CRGs in NB prognosis and TME. It is necessary to define cuproptosis-related biomarkers from the perspective of tumor immunity, which could help select potential candidates for immunotherapy. We submitted these genes into a Lasso penalized Cox regression analysis for establishing a five-cuproptosis-related gene signature and further established a risk scoring system. Here, all patients were separated into a high- and low-risk subgroup using this five-gene prognostic signature. These genes also showed differential expression in various characteristic groups of all three datasets and NB cell lines. *Via* PubMed, we found that these target genes play a crucial role in the prognosis and malignancy of tumors. Yuan et al. showed through a comprehensive pan-cancer analysis that LIAS may have potential significance in the progression of various cancers and also predict the efficacy of immunotherapy in cancer patients (43). PDHB could act as regulatory targets of multiple non-coding RNAs to regulate tumor cell progression (44–46). GLS is a key enzyme in glutamine metabolism with diverse functions in tumorigenesis (47). MTF1 is an important component of the metal regulatory system in mammalian cells, and the knockdown of MTF1 inhibits the epithelial-to-mesenchymal transition in ovarian cancer cells (48). PDHA1 was founded to be involved in the oncogenesis and progression of numerous malignancies through metabolic regulation (49, 50). In several tumor types such as hepatocellular carcinoma, cholangiocarcinoma, ovarian cancer, or esophageal squamous carcinoma, this gene regulates tumor progression by modulating the Warburg effect or metabolic reprogramming (50–55). Moreover, PDHA1 may be a prognostic and immune-related biomarker in a variety of cancers (56). Therefore, PDHA1 is considered a key target for anti-cancer therapy. Here, PDHA1 was thus selected for further expression analysis and functional validation in NB. The result revealed that this gene was specifically expressed in tissues and cells. Loss-of-function experiments indicated that PDHA1 silencing significantly suppressed the proliferation, migration, and invasion, in turn, promoted cell cycle arrest at the S phase and apoptosis of NB cells. Moreover, the model revealed a promising value in predicting both survival risk and clinicopathological features. Univariate and multivariate Cox regression analysis indicated that the riskscore was an independent prognostic indicator for NB patients, supporting it as a reliable predictive tool. Importantly, our findings obtained in the discovery cohort could be confirmed in the validation cohort. To provide clinicians with a quantitative approach to predicting the prognosis of NB patients, we integrated clinical characteristics with the signature to construct a combined nomogram model, which could more accurately predict their short-term and long-term survival.

Recent literature highlights the TIM as a complex environment in which the imbalances between tumor cells and the host immune response may result in a malignancy progression (57). Understanding the immunological status of the TME will allow us to deepen our knowledge of the anti-tumor immune response and develop more effective immunotherapies. Tumor-infiltrating immune cells are a critical part of the TME. Increasing evidence has revealed its clinicopathological significance in predicting prognosis and therapeutic response (58). We confirmed that the high-risk subgroup was significantly associated with characteristics related to the TME, especially immune infiltration. By investigating the level of immune cells infiltration in the TME, we observed two main features: 1) high-risk subgroup had lower immunoreactivity, 2) major lymphocyte subsets involved in anti-tumor immunity, including CD4^+^ memory T cells, CD4^+^ Tcm, CD8^+^ T cells, DCs, macrophages, mast cells, NKT cells, and Tregs were significantly absent in the high-risk subgroup. Hence, impaired anti-tumor immunity in high-risk patients might be the reason for their unfavorable prognosis. In general, the higher the anti-tumor immune cell infiltration the better the prognosis of patients. To verify this hypothesis, we performed a subgroup survival analysis of differentially presented TME scores and immune cell infiltration. As expected, patients with lower TME scores and tumor immune cell infiltration had a worse prognosis, and this finding was confirmed by two independent cohorts. We speculate that cuproptosis may follow a mechanism similar to ferroptosis and be involved in the regulation of anti-tumor immunity (59–61). These findings have contributed to the understanding of relationship between cuproptosis, TME, and NB. However, the exact mechanism is not clear.

In adult oncology, the study of the TIM has shown great promise in revealing new prognostic markers as well as new therapeutic forms and represents a significant advantage with the benefit of reduced toxicity over traditional chemotherapy. The introduction of immunotherapy into the field of pediatric oncology has been met with enthusiastic efforts, although with some delay. Immunotherapy is expected to become a promising choice for high-risk patients that were resistant to currently available therapies. On the other hand, the early and late toxicities of cytotoxic chemoradiotherapy caused serious problems in pediatric oncology, as it affected them until puberty and adulthood (62–64). Promisingly, immunotherapy offers a unique opportunity to create new treatment options that can be implemented into clinical practice. Further, the expression and regulation of immune checkpoint molecules also play a crucial surveillance role in the regulation of immune responses by inhibiting the activation of protective immune cells and promoting immune responses (65). High expression of immune checkpoint molecules generally benefits more from ICB therapy. In NB, the application of the anti-GD2 antibody Dinutuximab to the standard of care significantly improved the prognosis of patients. Indeed, Dinutuximab-dependent cytotoxicity was considered to be mediated primarily by neutrophils and NK cells (66, 67). The 5-year survival rate for patients with high-risk NB remains below 50%, which has triggered research into new immunotherapeutic approaches. ICB therapies have proven highly successful in a variety of adult tumors, but challenges remain in pediatric oncology. NB exhibits low immunogenicity due to its low mutational load and lack of MHC-I expression (68–70). In addition to low tumor immunogenicity, TIL responsiveness to NB may be heavily modulated by the presence of immune checkpoint molecules in TME. Although NB has been considered an immunologically ‘cold’ tumor (71, 72), a combination of different immunotherapies, as well as personalized strategies, may be promising ways. Our results revealed an excellent correlation between the signature and expression of immune checkpoint molecules. Combined with TIDE algorithm analysis, we explore the association between riskscore and ICB immunotherapy response in TARGET-NB cohort. TIDE results presented that more immunotherapeutic responders appeared in the low-risk subgroup than in the high-risk subgroup, which means low-risk patients with a lower TIDE score are more promising in responding to ICB. These results further suggest that the signature based on cuproptosis-related genes could help predict patients’ outcomes and identify optimal candidates for immunotherapy.

We believe that stratification of patients based on the established prognosis signature might prove useful. We, therefore, explored the downstream mechanism involved in the different riskgoup and showed that the phenotypic regulation of the cell-fate decisions in high- and low-risk subgroups was potentially regulated *via* affecting pathways associated with immune activation, cell cycle, autophagy, apoptosis, and oncogenesis. Of these, the mTOR pathway is an important pro-survival signaling pathway that is activated in most NB and is involved in regulating the protein levels of MYCN (73–76). AZD8055, a dual inhibitor of mTORC1-mTORC2, has been evaluated in preclinical NB models (77). In addition, targeting MEK1/2 inhibitors inhibited the growth of NB tumor cells (78). In contrast to many other tumors, NB typically has intact wild-type p53 (79, 80). Targeted antagonists against this pathway have shown promising results (81–83). In addition, inhibitors targeting apoptosis and autophagy have been tested *in vitro* and *in vivo* models (84, 85). Several drugs have been reported to induce apoptosis in neuroblastoma cells by increasing intracellular copper levels, demonstrating the therapeutic potential of copper-dependent pathways (16, 18). Thus, the relevant potential drugs were also predicted based on the significantly differentially expressed genes between the two risk subgroups. In some cases, small molecule drugs target both tumor cells and immune cells and exert beneficial or reversing effects on the immunosuppression of TME. A recent study confirmed that tipifarnib-mediated inhibition of small cell extracellular vesicle secretion may serve as a viable therapeutic strategy to enhance the anti-tumor efficacy of anti-GD2 immunotherapy in patients with high-risk NB (86). Several other small molecule drugs, including acarbose (87–89), brivanib (90–97), tipifarnib (98–106), fraxetin (107–109), NU1025 (110–112), trifluridine (113–117), imatinib (108, 118), quizartinib (119), lapatinib (120–122) have been tested in clinical trials in a variety of adult tumors and have shown promising results. However, children are not ‘small adults’. Pediatric tumors are likely to follow a unique immuno-oncologic mechanism. Further investigation of these issues will help in understanding molecular mechanisms leading to immune evasion in NB and provide a rational basis for novel therapies in the future. These areas remain to be explored.

To our knowledge, this is the first study involving the cuproptosis, tumor immunity, prognosis, and functional experiments. The major conclusions were validated in three independent and distinct cohorts of NB, a major strength of the study. The present results can contribute to improving the clinical risk evaluation of NB patients and offer new perspectives for future research on neoadjuvant therapy. However, some limitations ought to be considered in generalizing the present study’s findings. Firstly, this study is a retrospective review of public datasets; selection bias is inherent to the design. Thus, large and longitudinal prospective studies will be necessary to test this hypothesis before it can be implemented in clinical practice. Secondly, since immunotherapy has not been widely developed in NB, the patients’ response to immunotherapy was predicted by TIDE analysis. Finally, although functional analysis revealed the correlation between CRGs and immune-related features, the exact mechanism remains to be explored. Overall, additional research should be developed to clarify these hypotheses that hold the promise of improving the prognosis of NB patients.

In summary, we identified clues that suggest that CRGs affect the immune status and thus the prognosis of NB. The signature can help risk-adjusted personalized treatment and identify optimal candidates for immunotherapy. These findings highlight the crucial clinical implications of CRGs and help provide new insights into the molecular mechanisms of NB progression, as well as explore potential targeted therapies for NB patients.

## Data availability statement

The datasets presented in this study can be found in online repositories. The names of the repository/repositories and accession number(s) can be found in the article/**Supplementary Material**.

## Ethics statement

The studies involving human participants were reviewed and approved by the ethic committee of Children’s Hospital of Chongqing Medical University. Written informed consent to participate in this study was provided by the participants’ legal guardian/next of kin.

## Author contributions

FL, X-MT, and G-HW contributed to conception and design; FL and GHW contributed to administrative support; TM, CZ, and Z-XZ contributed to collection and processing of data; X-MT, J-KW, QL, and Y-HY contributed to data analysis and interpretation; X-MT, BX, CZ, and J-KW contributed to bioinformatics analysis; X-MT, J-KW, TM, and BX contributed to preparing all the figures and tables. X-MT, TM, BX, M-LC, and L-MJ contributed to the experimental process. X-MT and BX contributed to manuscript writing. All authors contributed to manuscript revision, read, and approved the submitted version.

## Funding

This work was supported by the Natural Science Foundation of Chongqing (cstc2021jcyj-msxmX0345), the Medical Scientific Research Project of Chongqing (NO.2022GDRC009), the general project of clinical medicine research of Children’s Hospital of Chongqing Medical University (NCRC-2019-GP-08).

## Acknowledgments

We gratefully thank Li-Jian Chao at the Chongqing Key Laboratory of Pediatrics for providing experiment assistance.

## Conflict of interest

The authors declare that the research was conducted in the absence of any commercial or financial relationships that could be construed as a potential conflict of interest.

## Publisher’s note

All claims expressed in this article are solely those of the authors and do not necessarily represent those of their affiliated organizations, or those of the publisher, the editors and the reviewers. Any product that may be evaluated in this article, or claim that may be made by its manufacturer, is not guaranteed or endorsed by the publisher.

## Abbreviations

NB, Neuroblastoma; CRGs, Cuproptosis-related genes; PCA, Principal component analysis; CSVA, Gene set variation analysis; ROC, Receiver operating characteristic; AUC, Area under the curve; TIM, Tumor immune microenvironment; TIDE, Tumor Immune Dysfunction and Exclusion.
