# Supplementary figures and images for: A novel cuproptosis-related subtypes and gene signature associates with immunophenotype and predicts prognosis accurately in neuroblastoma
Source: Front Immunol. 2022 Sep 23;13:999849. doi: 10.3389/fimmu.2022.999849 (PMC9540510; doi:10.3389/fimmu.2022.999849)

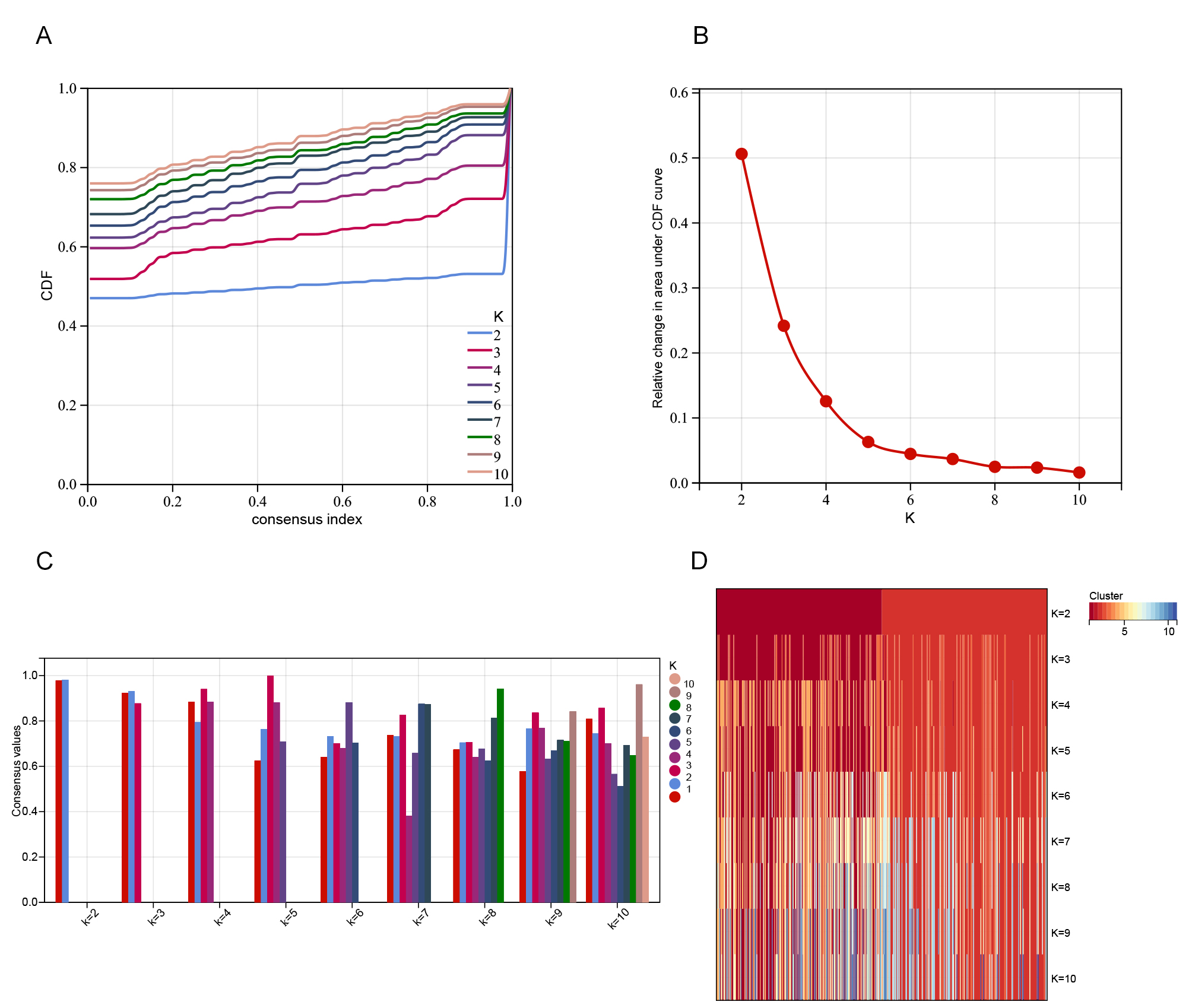

Supplement: Supplementary file 1 [file Image_1.jpeg]

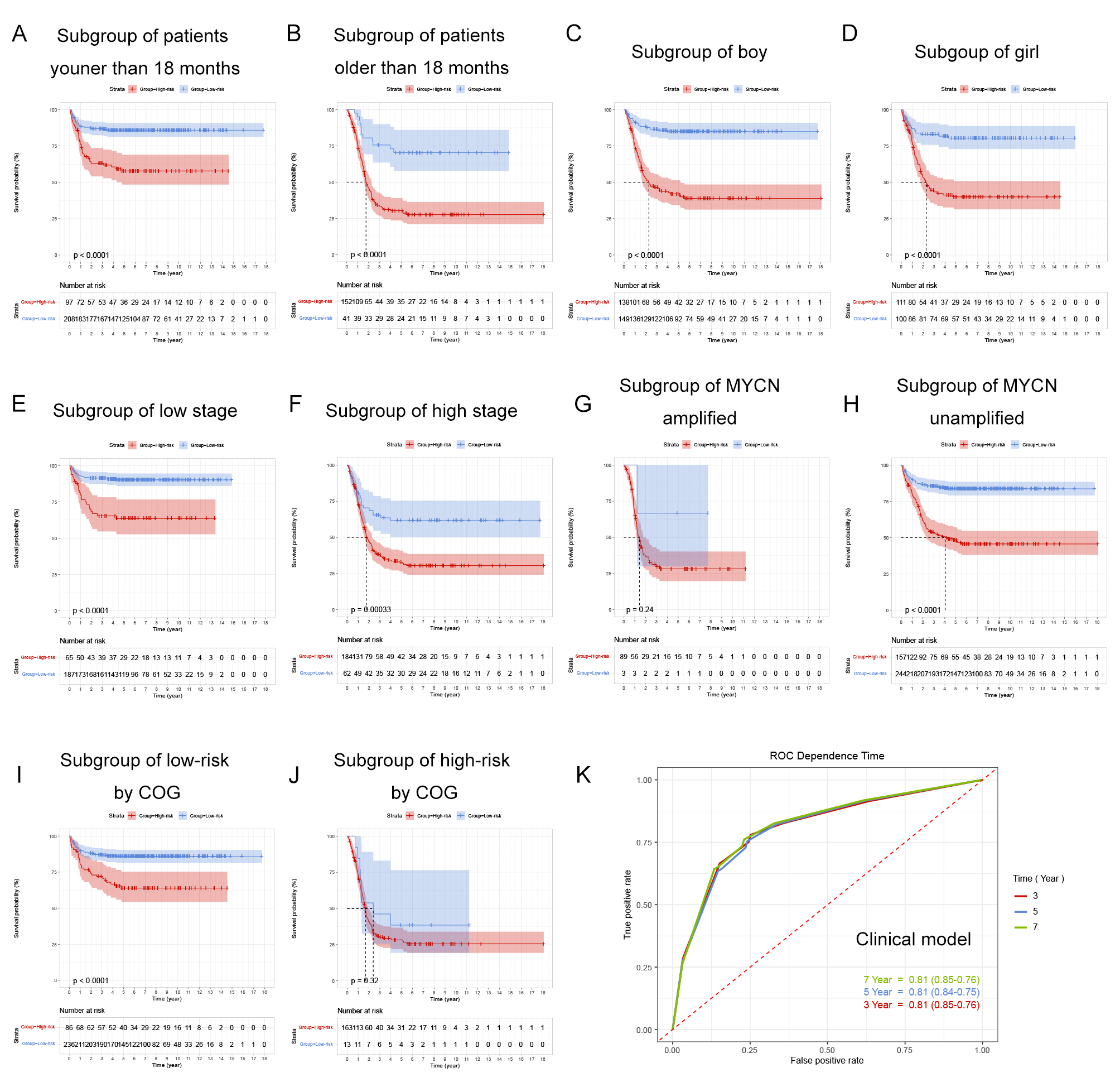

Supplement: Supplementary file 2 [file Image_2.jpeg]

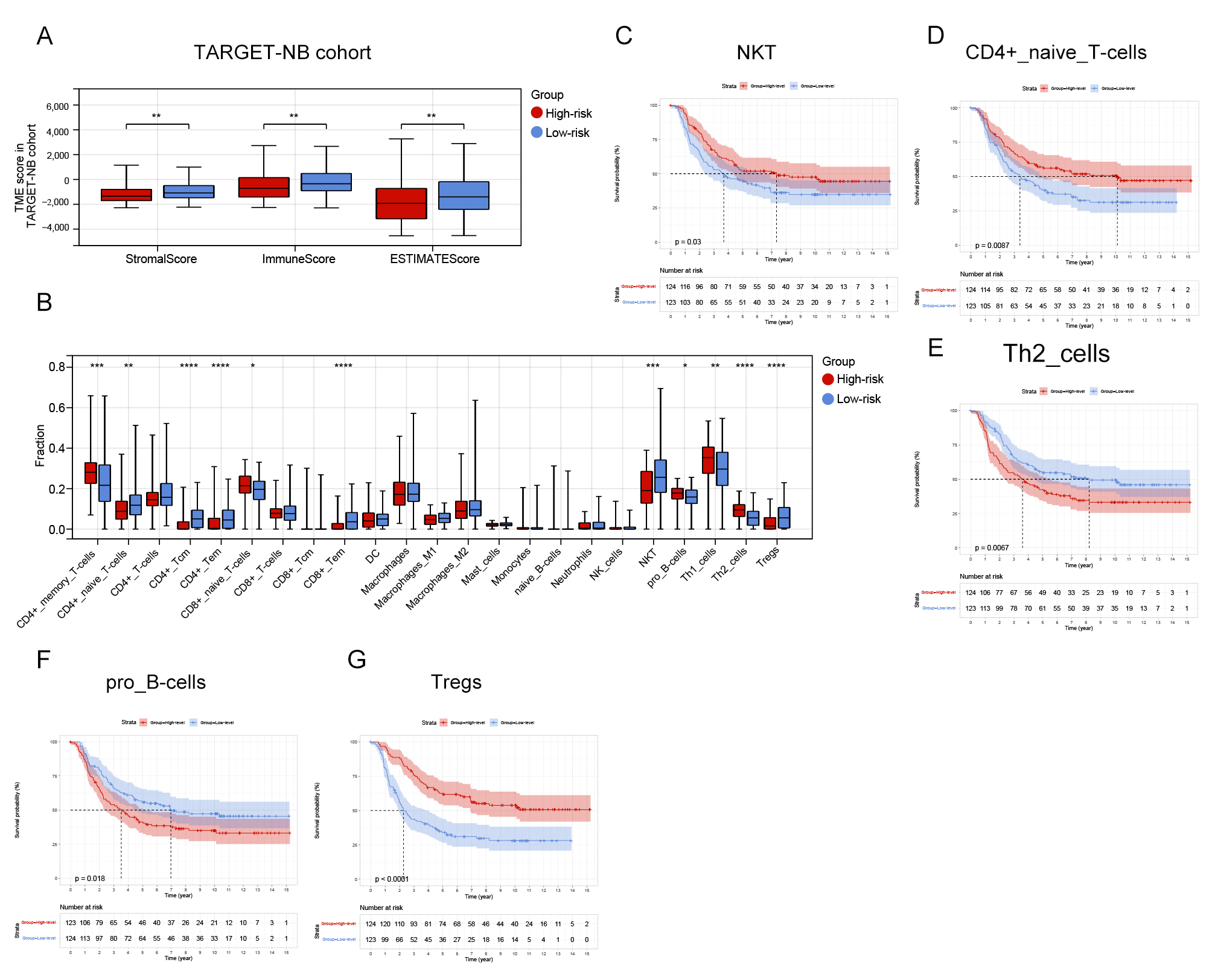

Supplement: Supplementary file 3 [file Image_3.jpeg]

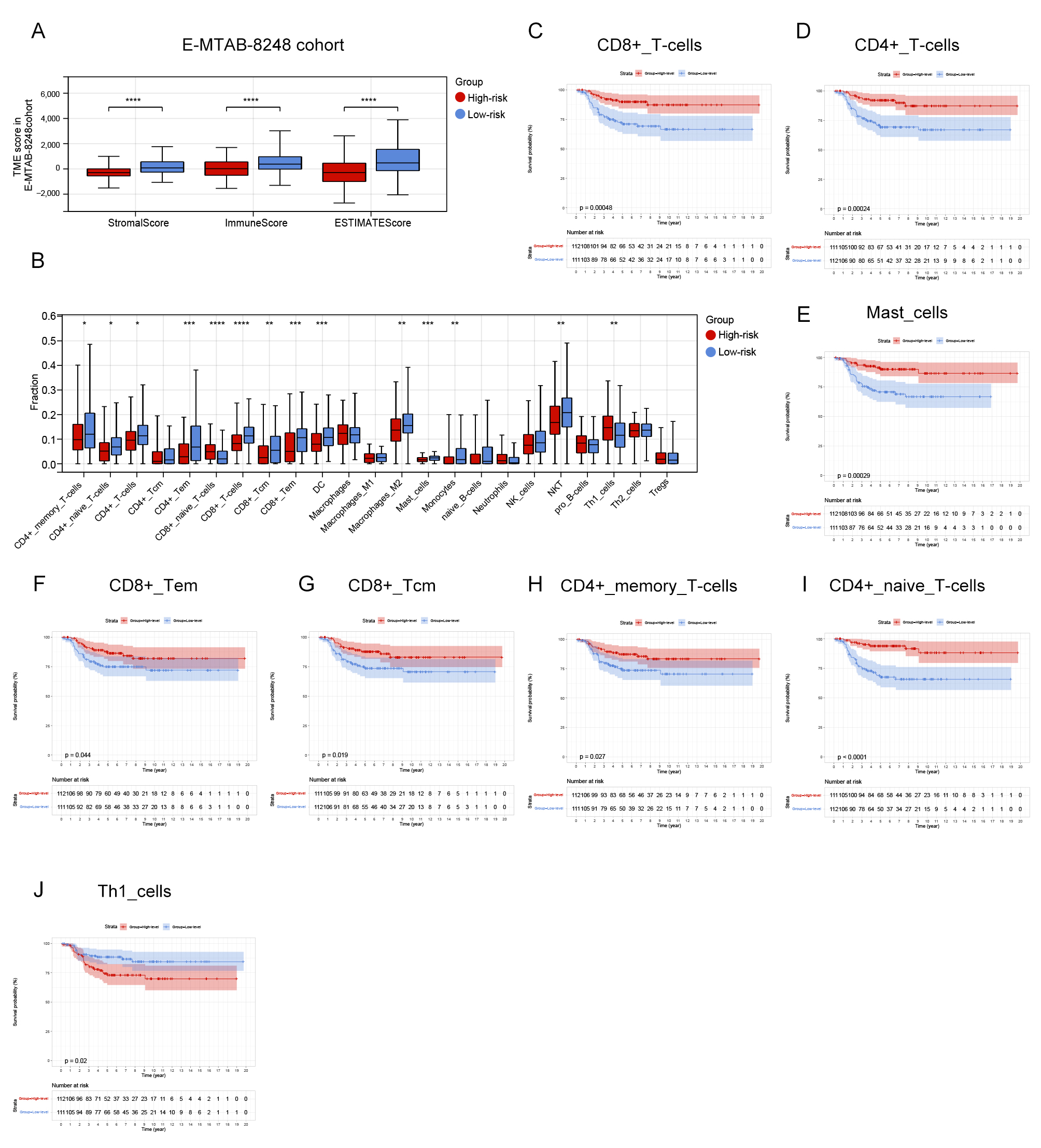

Supplement: Supplementary file 4 [file Image_4.jpeg]

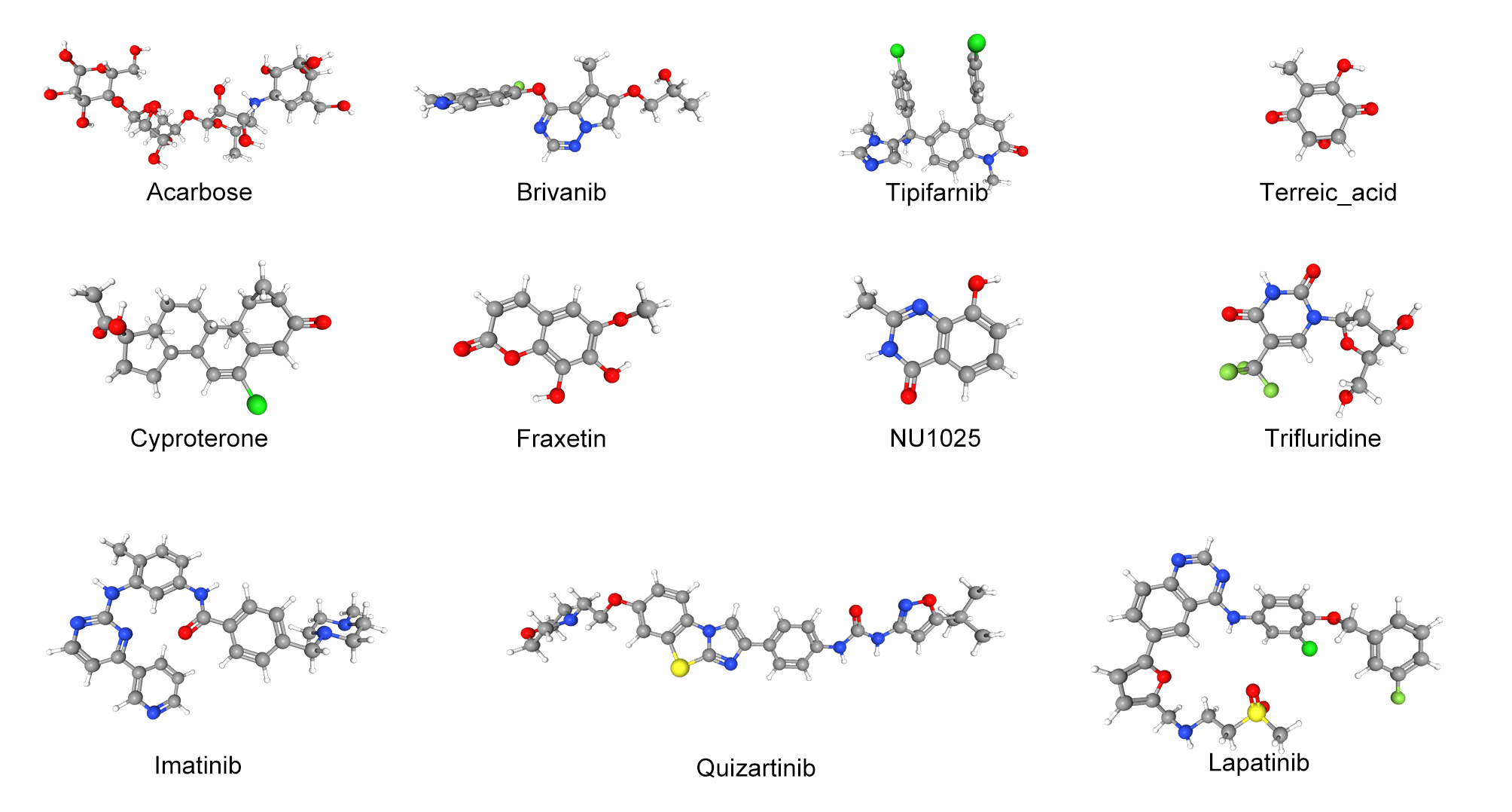

Supplement: Supplementary file 5 [file Image_5.jpeg]

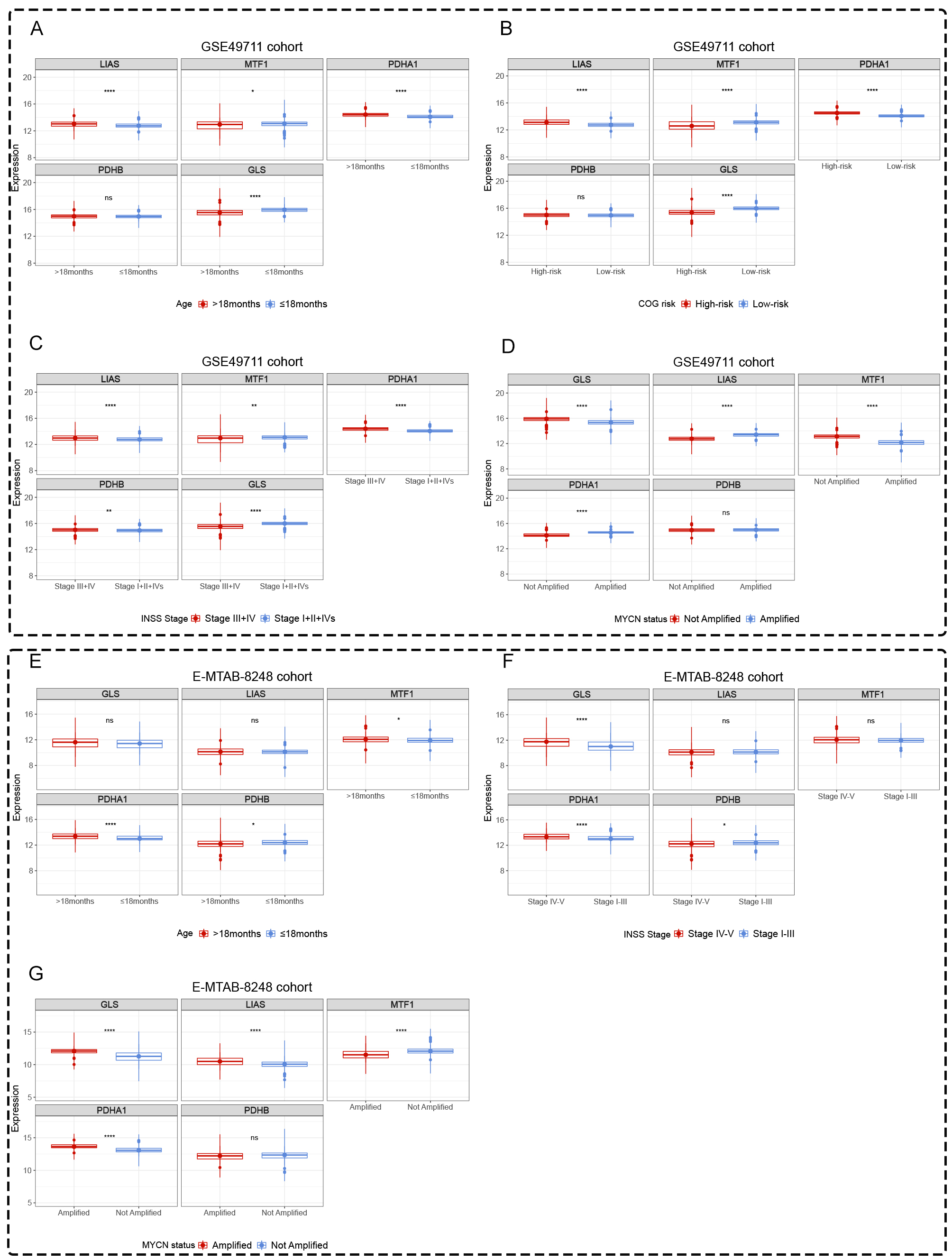

Supplement: Supplementary file 6 [file Image_6.jpeg]

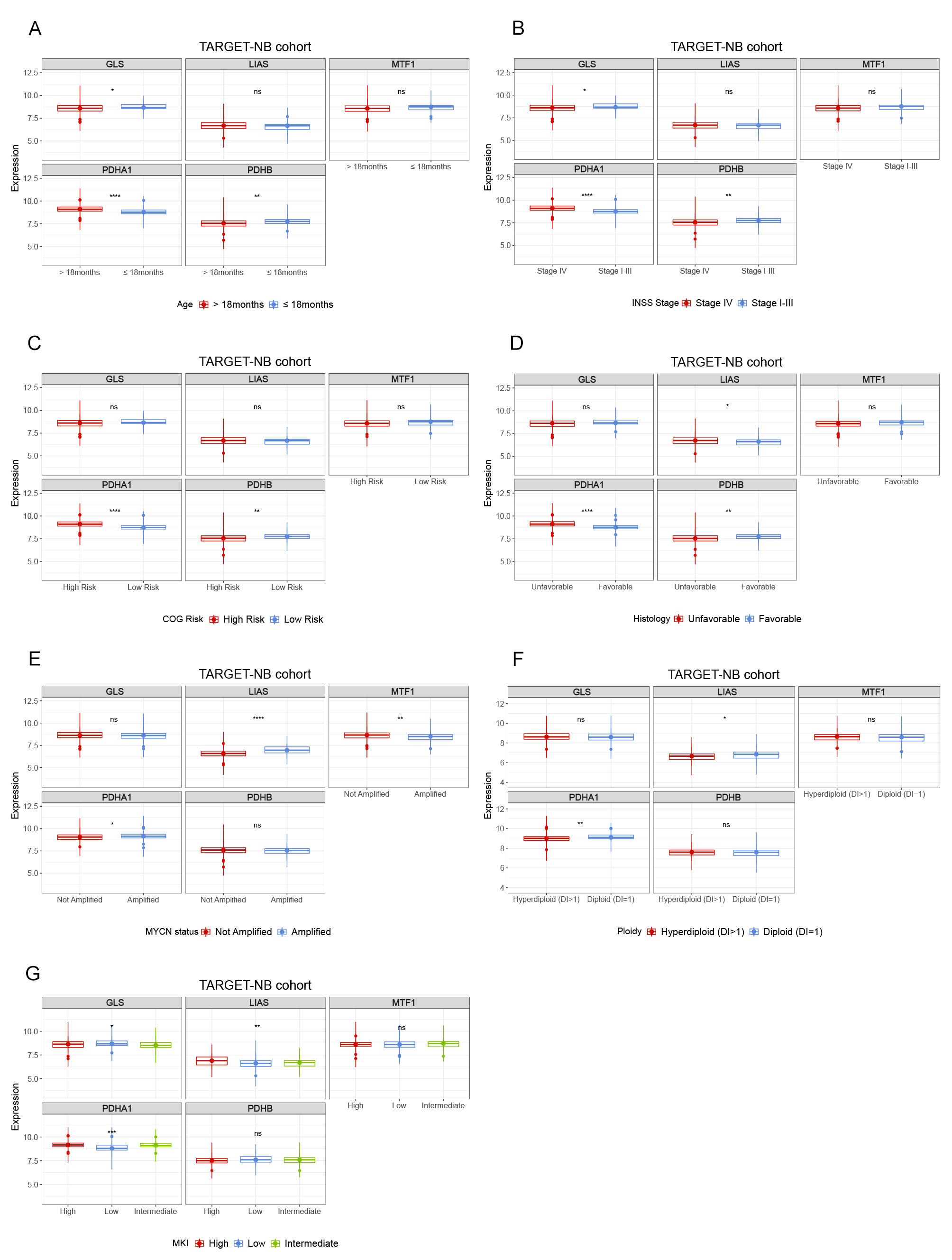

Supplement: Supplementary file 7 [file Image_7.jpeg]
