# Supplementary material for: A novel cuproptosis-related subtypes and gene signature associates with immunophenotype and predicts prognosis accurately in neuroblastoma
Source: Front Immunol. 2022 Sep 23;13:999849. doi: 10.3389/fimmu.2022.999849 (PMC9540510; doi:10.3389/fimmu.2022.999849)
Supplement: Supplementary file 12 [file Table_5.docx]

**Supplementary Table 5**

**A**

**Primers used for quantitative real time PCR**

| **RNA** | **Forward primer** | **Reverse primer** |
| --- | --- | --- |
| MTF1 | AGCACAATTTCACCAGCAATCAT | TGACTGACTCTGCATCACCATTAA |
| GLS | ACCAAAGTTCCCTTCTGTCTTCA | TGAAGTCACAACAATTGCTCCAG |
| PDHB | GGATAGAGGACACGACCAAGATG | GCTCCTCATCCATACCCTGATTT |
| PDHA1 | AGGGCCAGATATTCGAAGCTTAC | AGGAATGAAATCGCCTCTCTTGT |
| LIAS | AGTATGTGAGGAAGCTCGATGTC | GGAGGAGGATTTCTTGCAGTCTTA |
| GAPDH | CCTTCCTGGGCATGGAGTC | TGATCTTCATTGTGCTGGGTG |

**B**

**Three siRNA sequences were used to silence the PDHA1 gene**

| **NAME** | **Sequence (5’ - 3’)** |
| --- | --- |
| Si-PDHA1-1-ss | GCAGAGCUUACAGGACGAATT |
| Si-PDHA1-1-as | UUCGUCCUGUAAGCUCUGCTT |
| Si-PDHA1-2-ss | CCAGUGUGGAAGAACUAAATT |
| Si-PDHA1-2-as | UUUAGUUCUUCCACACUGGTT |
| Si-PDHA1-3-ss | GAGAAUAAUCGCUAUGGAATT |
| Si-PDHA1-3-as | UUCCAUAGCGAUUAUUCUCTT |
